# Diagnostic and Prognostic Properties of Osteoprotegerin in Patients with Acute Dyspnoea: Observations from the Akershus Cardiac Examination (ACE) 2 Study

**DOI:** 10.1371/journal.pone.0160182

**Published:** 2016-07-27

**Authors:** Ragnhild Røysland, Mohammed Osman Pervez, Marit Holmefjord Pedersen, Jon Brynildsen, Arne Didrik Høiseth, Tor-Arne Hagve, Helge Røsjø, Torbjørn Omland

**Affiliations:** 1 Division of Medicine, Akershus University Hospital, Lørenskog, Norway; 2 Institute of Clinical Medicine, University of Oslo, Oslo, Norway; 3 Section for Medical Biochemistry, Division for Diagnostics and Technology, Akershus University Hospital, Lørenskog, Norway; Scuola Superiore Sant'Anna, ITALY

## Abstract

**Background:**

Circulating osteoprotegerin (OPG) levels are increased in patients with chronic heart failure (HF). The diagnostic and prognostic merit of OPG measurement in patients admitted with acute dyspnoea is unknown.

**Objectives:**

To evaluate the diagnostic and prognostic value of measuring OPG in patients admitted to hospital with acute dyspnoea.

**Methods:**

OPG was analysed by ELISA in 308 patients admitted due to acute dyspnoea. Investigators blinded to OPG results adjudicated the diagnosis for the index hospitalization. Clinical outcomes were obtained from hospital records.

**Results:**

In total, 139 patients (45%) were hospitalized with acute HF. OPG levels on hospital admission were higher in patients with acute HF vs. no acute HF, 7.8 (5.5–10.4) vs. 5.4 (3.8–7.2) pmol/L, p<0.001. The area under the receiver operator characteristic curve (ROC AUC) of OPG to discriminate between HF vs. non-HF was 0.695 [95% CI 0.636–0.754]. OPG did not provide incremental information to the ED physician’s prediction or N-terminal pro-B-type natriuretic peptide regarding the diagnosis of acute HF. OPG levels (log transformed) were associated with mortality in crude analysis (HR (95% CI) 1.87 (1.34 to 2.61), p<0.001), but this association was attenuated and no longer significant after including established cardiac biomarkers into the model.

**Conclusion:**

In patients admitted to hospital with acute dyspnoea, OPG levels are higher in patients with acute HF than in those with dyspnoea from other causes. However, OPG does not provide incremental information beyond ED physician assessment for the diagnosis of acute HF or beyond clinical risk variables and established cardiac biomarkers concerning prognosis.

## Introduction

During the past decade, the B-type natriuretic peptides (e.g. BNP and NT-proBNP) have been recognized as important tools for emergency department physicians to differentiate between dyspnoea of cardiac origin and dyspnoea from other causes [1]. Although natriuretic peptides are accurate tools when levels are clearly elevated or low, there is a large grey zone in which the diagnostic accuracy is relatively modest. Accordingly, the search for additional diagnostic tools to accurately diagnose HF in patients with acute dyspnoea is still considered a high priority [2]. Inflammation plays an important role in the pathogenesis of heart failure [3], and circulating markers of inflammation have therefore been proposed as potential biomarkers in heart failure.

OPG is a cytokine in the tumour necrosis factor receptor superfamily. In bone metabolism OPG plays an important role as a protector of bone degradation. However, OPG has also been linked to cardiovascular disease [4] and to the development of heart failure after myocardial infarction [5]. The OPG/RANKL/RANK system’s ability to stimulate matrix metalloproteinase (MMP) activity, and thereby influence extracellular matrix degradation, has been proposed as one mechanism explaining how this system might be linked to heart failure development [6].

In patients with chronic heart failure, OPG levels are associated with mortality in patients with systolic heart failure of any cause [7], and with the worsening and progression of heart failure in patients with systolic heart failure of ischemic origin [8]. In a recent systematic review of biomarkers of heart failure after acute myocardial infarction, OPG was found to be important for risk assessment [9]. The diagnostic and prognostic value of measuring OPG in the setting of acute heart failure, however, has been less well studied, and whether OPG is useful as a diagnostic tool for identifying patients with acute heart failure as the cause of acute dyspnoea is not known. Accordingly, we hypothesized that OPG provides incremental information to established risk indices for diagnosis and prognosis in acute HF.

## Materials and Methods

### Patients—Akershus Cardiac Examination (ACE) 2 Study

The design and methodology of the Akershus Cardiac Examination (ACE) 2 Study have been described previously [10]. In brief, 314 consecutive patients admitted with acute dyspnoea to the emergency department at Akershus University Hospital were included. Serum samples were available from 312 patients for this sub-study, but 4 patients were excluded due to technical difficulties with the OPG measurements, leaving 308 patients in the final cohort.

Eligibility criteria for the study were age ≥18 y, dyspnoea considered as the primary cause for hospitalization by the physician examining the patient in the Emergency Department (ED), and the time from hospital admission to study inclusion <24 h. Exclusion criteria were dementia or other cause precluding informed patient consent; disseminated malignant disease; a history of acute myocardial infarction (AMI), coronary intervention, or major surgery within the last 2 weeks; or inadequate blood sampling. Patients were included from Monday through Thursday, from 8.00 a.m. to 2 p.m., from June 2009 to November 2010. The study was conducted according to the Declaration of Helsinki, approved by the Norwegian Regional Committees for Medical and Health Research Ethics (REC) South East (#5.2008.2832) and Akershus University Hospital, and all patients provided written informed consent before study commencement.

### Data collection

Dedicated personnel collected clinical information by interviewing the emergency department (ED) physician or directly from the patient. This information was also checked against the patient’s medical records. An estimate of the probability of acute HF as the primary cause of dyspnoea on a scale from 0% (highly unlikely) to 100% (very likely) was obtained from the ED physician. Blood pressure, heart rate, and body temperature on hospital admission were collected from the patient records. We defined history of coronary artery disease (CAD) as previous myocardial infarction, previous coronary intervention (percutaneous or coronary artery by-pass grafting) or more than 50% stenosis on coronary angiography. Left ventricular ejection fraction (LVEF) was determined by echocardiography as part of clinical routine, and was not available for all the patients.

### Adjudication of diagnosis and follow-up data

The final diagnoses of the index hospitalizations were determined by two independent senior physicians, who reviewed all medical records, including follow-up data, but were blinded to OPG data. Index hospitalization diagnoses were concordant in 95% of the cases. Discrepancy was resolved by consensus. HF was based on the criteria proposed by the European Society of Cardiology requiring typical signs and symptoms of HF and objective evidence of structural or functional myocardial abnormality [11]. In patients with preserved EF (LVEF≥50%), the diagnosis of HF was based on echocardiographic indices associated with diastolic dysfunction (e.g. left atrial enlargement, LV hypertrophy, inverted E/A ratio, etc). Chronic obstructive pulmonary disease (COPD) was diagnosed according to established GOLD criteria [12]. Survival status on November 1^st^, 2012 was obtained from electronic hospital records, which are synchronized with Statistics Norway on a monthly basis.

### Biochemical analysis

After admission to hospital, blood samples were obtained within 24 h. A second set of blood samples were acquired from patients staying in hospital for 24–48 hours, drawn approximately 24 h after the first set of samples. We also obtained blood samples in a subset of patients on the day of discharge in patients staying longer than 48 h. OPG levels in serum were measured with a commercially available sandwich ELISA (BI-20403, Biomedica, Vienna, Austria). The assay measures both free OPG and complexed OPG-RANKL. According to the manufacturer, the assay detects both the monomeric and the dimeric form of OPG. The limit of detection for the assay is 0.07 pmol/L, the intra-assay precision is ≤ 3%, and the inter-assay precision is ≤ 5%. NT-proBNP and high sensitivity troponin T (hs-cTnT) concentrations were measured in samples obtained at the same time points as OPG by the proBNP II assay and the STAT hs Troponin T assay, respectively, on a Cobas Platform (Roche Diagnostics, Penzberg, Germany). CRP and creatinine in serum were determined on hospital admission by standard biochemical methods and creatinine clearance was calculated by the Cockcroft-Gault formula [13].

### Statistics

Variables are presented as means (standard deviation) for continuous normally distributed variables and as median (25–75 percentile) for non-normal variables. Categorical variables are presented as proportions. Normally distributed continuous variables were compared using the Student’s *t*-test and non-normally distributed variables compared by the Mann-Whitney-U test. Proportions were compared using the Chi-square-test. Correlations were assessed by Spearman rank correlation and variables associated with OPG levels were examined by linear regression analysis. The association between OPG levels and the diagnosis of heart failure was examined by logistic regression analysis. Diagnostic accuracy was evaluated by assessing the receiver-operating characteristics (ROC) curve. Results are presented in a figure, as well as numerically as area under the ROC curve (AUC) with 95% CI. We assessed the prognostic value of OPG by Cox proportional hazard regression analyses, and variables that were associated with mortality by univariable analyses were included in the multivariable models. OPG levels and other non-normally distributed variables were transformed by the natural logarithm prior to regression analyses. P-values <0.05 were considered statistically significant. Statistical analyses were performed with the statistical programming language R (R Development Core Team, 2008).

## Results

### Patient characteristics

In the entire cohort of 308 patients with acute dyspnoea, 139 patients were classified as hospitalized due to acute heart failure. Of these, 83 patients (60%) had a previous history of HF, while 56 patients (40%) were considered to have *de novo* acute heart failure. The median level of OPG in the entire cohort (n = 308) was 6.2 (4.6–9.1) pmol/L.

Characteristics of the patients according to presence (n = 139) or absence (n = 169) of acute HF are show in Table 1. Patients with acute HF were older, more likely to be male, have decreased kidney function and have a history of CAD, hypertension and diabetes. OPG levels were 7.8 (5.5–10.4) pmol/L and 5.4 (3.8–7.2) pmol/L in patients with and without acute HF, respectively (p<0.001).

**Table 1:** Baseline characteristic according to HF diagnoses.

	No acute HF	Acute HF	*P*
N	169	139	
OPG baseline (pmol/L)	5.4 [3.8, 7.2]	7.8 [5.5, 10.4]	<0.001
OPG at day 2 (pmol/L)	5.6 [4.3, 7.7]	7.4 [5.3, 10.6]	<0.001
OPG at discharge (pmol/L)	5.2 [3.82 6.9]	8.7 [6.3, 11.3]	<0.001
***Demographics***			
Age (years)	66 (15)	75 (11)	<0.001
Female/male	97/72 (57/43)	53/86 (38/62)	0.001
Body mass index (kg/m^2^)	26.50 (8.0)	26.55 (5.9)	0.954
Current smoker	55 (32.5)	30 (21.6)	0.044
Creatinine Clearance (mL/min)	79 [63, 103]	59 [41, 82]	<0.001
NYHA 4	69 (41)	62 (45)	0.581
LVEF (%)	60 [50, 60]	40 [30, 55]	<0.001
HF type			
HF, systolic dysfunction (%)	-	87 (63)	
HF, preserved ejection fraction (%)	-	52 (37)	
Physician assessment of HF (%)	20 [10, 30]	60 [30, 80]	<0.001
***History of***			
Heart failure (%)	14 (8)	83 (60)	<0.001
Hypertension (%)	49 (29)	67 (48)	0.001
Coronary artery disease (%)	32 (19)	74 (53)	<0.001
Atrial fibrillation (%)	27 (16)	66 (48)	<0.001
Diabetes mellitus (%)	24 (14)	41 (30)	0.002
COPD (%)	93 (55)	59 (42)	0.037
Asthma (%)	22 (13)	6 (4)	0.009
***Medication***			
Beta blocker (%)	50 (30)	85 (61)	<0.001
ACEI or ARB (%)	51 (30)	83 (60)	<0.001
Loop diuretics (%)	46 (27)	93 (67)	<0.001
Statin (%)	48 (28)	76 (55)	<0.001
Aldosterone antagonist (%)	7 (4)	21 (15)	0.001
***Biomarkers***			
CRP (mg/L)	22 [5, 60]	13 [5, 32]	0.03
hs-cTnT (ng/L)	12.8 [4.2, 25.1]	37.8 [22.1, 74.1]	<0.001
NT-proBNP (ng/L)	347.7 [119.1, 1027.6]	3576.0 [1643.5, 8262.9]	<0.001

HF heart failure; OPG osteoprotegerin; IQR interquartile range; NYHA New York Heart Association; LVEF left ventricular ejection fraction; COPD chronic obstructive pulmonary disease; ACEI angiotensin converting enzyme inhibitor; ARB angiotensin receptor blocker; CRP c-reactive protein; hs-cTnT high sensitive cardiac troponin T; NT-proBNP N-terminal pro b-type natriuretic peptide; Data are presented as median [25–75 percentile], mean (standard deviation) or number (%).

OPG at baseline correlated with age (ρ = 0.47; p < 0.001), creatinine clearance (ρ = 0.47; p < 0.001) and with the biomarkers NT-proBNP (ρ = 0.55; p < 0.001), hs-cTnT (ρ = 0.48; p < 0.001), and CRP (ρ = 0.30; p < 0.001). The factors associated with higher OPG levels by multivariable analysis were age, NT-proBNP and CRP, whereas a history of COPD was associated with lower OPG levels (Table 2).

**Table 2:** Variables associated with OPG levels on study inclusion.

	Univariable analysis			Multivariable analysis^a^			Multivariable analysis^b^		
	Coefficient (β)	SE (β)	*P*	Coefficient (β)	SE (β)	*P*	Coefficient (β)	SE (β)	*P*
***Demografics*:**									
Age	0.017	0.002	<0.001	-	-	-	0.006	0.002	0.004
Gender	0.102	0.06	0.103	0.120	0.06	0.032	0.034	0.05	0.545
BMI	-0.007	0.004	0.104	-0.0003	0.004	0.937			
Ln Creatinine	-0.513	0.08	<0.001	-0.363	0.07	<0.001	0.121	0.08	0.138
***History of*:**									
Diabetes mellitus	0.197	0.08	0.010	0.182	0.07	0.008	0.049	0.06	0.442
Hypertension	0.161	0.06	0.012	0.075	0.06	0.203			
Coronary artery disease	0.163	0.07	0.013	0.074	0.06	0.219			
Heart failure	0.282	0.07	<0.001	0.157	0.06	0.012	-0.073	0.06	0.259
COPD	-0.110	0.06	0.077	-0.177	0.06	0.002	-0.151	0.05	0.005
Atrial fibrillation	0.304	0.07	<0.001	0.159	0.06	0.013	0.003	0.06	0.961
***Biomarkers*:**									
Ln CRP	0.094	0.02	<0.001	0.072	0.02	<0.001	0.065	0.01	<0.001
Ln NT-proBNP	0.164	0.01	<0.001	0.134	0.02	<0.001	0.100	0.02	<0.001
Ln hs-cTnT	0.219	0.02	<0.001	0.154	0.03	<0.001	0.052	0.03	0.092

OPG osteoprotegerin; BMI body mass index; COPD chronic obstructive pulmonary disease; prefix ln for variables transformed by natural logarithm; CRP c-reactive protein; hs-cTnT high sensitive cardiac troponin T; NT-proBNP N-terminal pro b-type natriuretic peptide;

^a^Adjusted for age

^b^Adjusted R^2^ = 0.41;

Model includes all variables significantly associated with OPG when adjusting for age.

### Serial OPG levels during the index hospitalization

**Fig 1:**
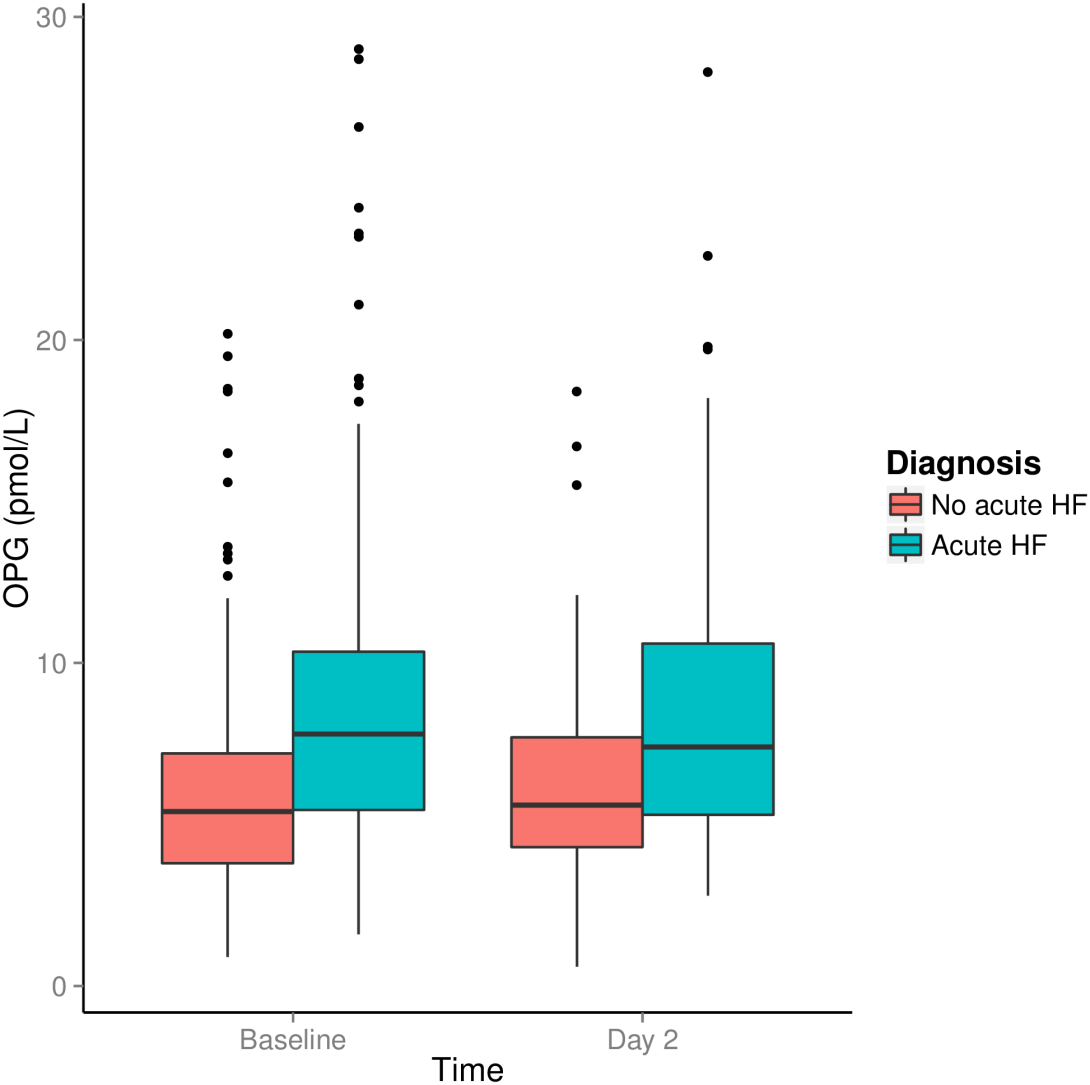
Median OPG level at baseline and day 2 for patients with and without heart failure. The bars represent the interquartile range (25–75 percentile). The whiskers represent the minimum and maximum values that are not outliers. The dots represent outliers, which are values between 1.5 and 3 times the interquartile range.

In 228 patients, OPG measures were available on day 2 of the hospital stay. For patients staying longer than 2 days, discharge OPG levels were available in 95 patients. Median (Q1-3) OPG levels decreased slightly from baseline to day 2 [ΔOPG -0.2 (-0.9–0.5) pmol/L, p = 0.043], but baseline and discharge levels were not significantly different [ΔOPG -0.2 (-1.3–0.9) pmol/L, p = 0.188] in the cohort as a whole. OPG levels obtained at baseline and day 2 stratified according to diagnosis of acute HF are shown in Fig 1. In patients with acute HF, OPG levels were higher at baseline (n = 139), at day 2 (n = 103) and at discharge (n = 45) than in patients with non-HF related dyspnoea [7.8 (5.5–10.4) vs. 5.4 (3.8–7.2) pmol/L, p<0.001; 7.4 (5.3–8.6) vs. 5.6 (4.3–7.7) pmol/L, p<0.001; 8.7 (6.3–11.3) vs. 3.8 (5.2–6.9) pmol/L, p<0.001]. Throughout the hospital stay, OPG levels were unchanged in patients with acute HF in whom OPG measurement was available at discharge (n = 45). In the patients with non-HF related acute dyspnoea, OPG levels decreased transiently from baseline to day 2 (ΔOPG -0.2 (-0.9–0.4) pmol/L; p = 0.029), while the change from baseline to discharge was not significant (p = 0.133). In comparison, there was a significant decrease in levels of NT-proBNP from baseline to discharge in the entire cohort (n = 93; ΔNT-proBNP -281 (-1688-1275) pg/mL; p<0.001).

### Baseline OPG levels and acute HF diagnosis

As mentioned above, unadjusted OPG levels at baseline were significantly higher in patients classified as acute HF than those with another diagnosis. By multivariable analysis OPG levels remained associated with the diagnosis of acute HF after adjustment for clinical variables (Table 3). However, after including the biomarkers NT-proBNP and hs-cTnT in the model, OPG levels were no longer significantly associated with the acute HF diagnosis.

**Table 3:** Variables associated with the index diagnosis of acute heart failure.

	Univariable analysis			Multivariable analysis^a^			Multivariable analysis^b^		
	Coefficient (β)	SE (β)	*P*	Coefficient (β)	SE (β)	*P*	Coefficient (β)	SE (β)	P
***Demografics***									
Age	0.011	0.002	<0.001	0.003	0.002	0.137	-0.0002	0.002	0.919
Gender	0.191	0.06	0.001	0.065	0.05	0.188	0.027	0.05	0.556
BMI	0.000	0.00	0.954						
***History of*:**									
Diabetes mellitus	0.227	0.07	0.001	0.027	0.06	0.648	0.017	0.05	0.745
Hypertension	0.203	0.06	<0.001	0.123	0.05	0.011	0.067	0.04	0.119
Heart failure	0.590	0.05	<0.001	0.456	0.06	<0.001	0.342	0.06	<0.001
Coronary artery disease	0.376	0.06	<0.001	0.054	0.06	0.357	-0.018	0.05	0.732
COPD	-0.125	0.06	0.028	-0.117	0.05	0.013	-0.115	0.04	0.007
***Tests***									
Ln Creatinine-Clearance	-0.308	0.05	<0.001	-0.061	0.06	0.336	0.032	0.06	0.578
***Biomarkers***									
Ln OPG	0.300	0.05	<0.001	0.101	0.05	0.037	0.002	0.05	0.965
Ln NT-proBNP	0.159	0.01	<0.001				0.091	0.02	<0.001
Ln hs-cTnT	0.228	0.02	<0.001				0.103	0.03	<0.001
Ln CRP	-0.032	0.02	0.051				-0.053	0.01	<0.001

BMI body mass index; COPD chronic obstructive pulmonary disease; prefix Ln for variables transformed by natural logarithm; OPG osteoprotegerin; CRP c-reactive protein; hs-cTnT high sensitive cardiac troponin T; NT-proBNP N-terminal pro b-type natriuretic peptide;

^a^Multivariable model including clinical variables and OPG.

^b^Multivariable model including clinical variables and biomarkers in addition to OPG.

**Fig 2:**
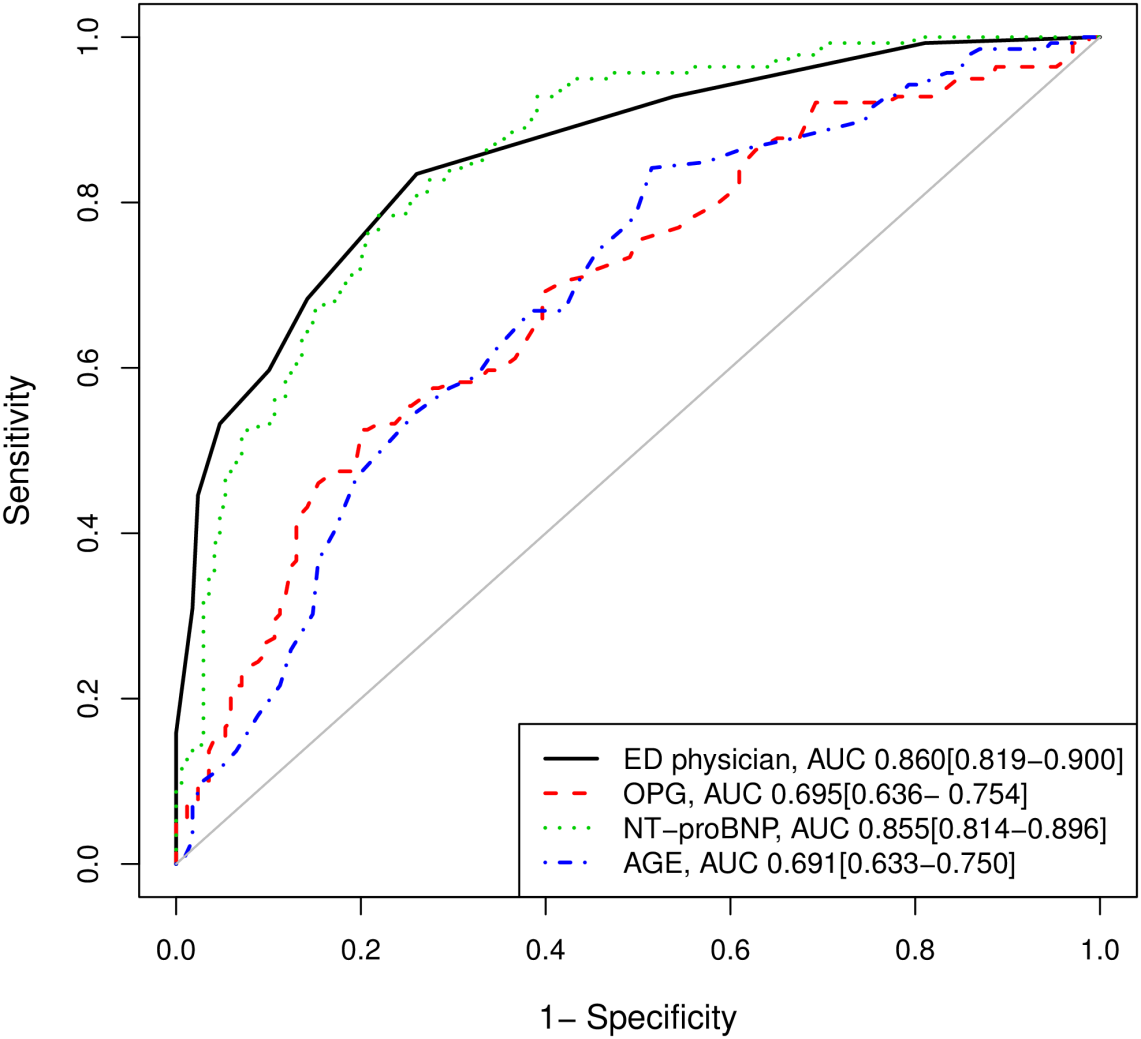
ROC curves for the prediction of the diagnosis of acute heart failure. The AUC with 95%CI at the right bottom are for each variable separately.

The overall accuracy of OPG, expressed as ROC-AUC, to discriminate between patients with acute HF vs. dyspnoea due to other causes (AUC: 0.695 [95% CI 0.636–0.754]) was lower than the AUC of NT-proBNP (AUC: 0.855 [95% CI 0.814–0.896]). As shown in Fig 2, NT-proBNP had a similar ability to predict acute HF as the ED physician (AUC: 0.860 [95% CI 0.819–0.900]), while OPG had similar ability to predict acute HF as knowing the patients age (AUC: 0.691 [95% CI 0.633–0.75]). Adding information about OPG levels to the ED physician prediction and NT-proBNP (AUC: 0.887 [95% CI 0.851–0.924]) did not significantly improve the prediction of acute HF (AUC: 0.888 [95% CI 0.852–0.924]; p = 0.21).

To assess the effect of coronary artery disease (CAD) on OPG concentrations, we also stratified patients with acute HF according to the presence (n = 74) or absence (n = 65) of a history of CAD. No significant difference was observed between patients with and without a history of CAD (8.5 (5.5–11.8) vs. 7.6 (5.5–9.5) pmol/L, p = 0.45).

### OPG and mortality

**Fig 3:**
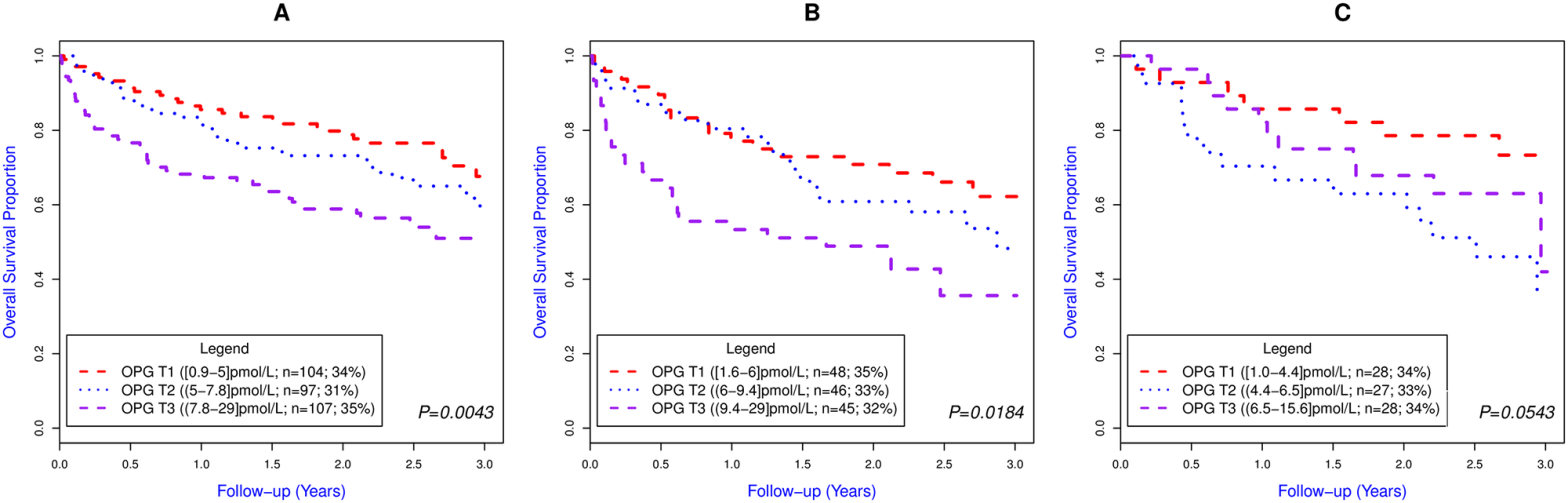
Kaplan Meier curves according to OPG tertile in patients with A) acute dyspnoea (n = 308) B) acute HF (n = 139) and C) acute exacerbation of COPD (n = 83). OPG T1, T2 and T3 refer to OPG tertile 1 (OPG ≤5.0 pmol/L], tertile 2 (OPG 5.0–7.8 pmol/L) and tertile 3 (OPG >7.8 pmol/L), respectively. The *P* values are for the log-rank-test.

During a median (Q1-3) follow-up of 817 (492–996) days, 112 patients (36%) died. The association between OPG tertiles and mortality is shown in Fig 3A (p = 0.0043 for log-rank test). Log-transformed OPG levels were associated with mortality in unadjusted Cox regression analysis (HR: 1.87 (1.34–2.61); p<0.001) (Table 4). In a multivariable Cox regression analysis that included age, BMI, blood pressure on admission, history of COPD, and the biomarkers NT-proBNP, CRP, and hs-cTnT, adding OPG provided no incremental prognostic information (Table 4).

**Table 4:** Cox models for mortality in patients with acute dyspnoea (n = 308).

	Univariable analysis^a^			Multivariable analysis^b^			Multivariable analysis^c^		
	HR	95% CI	*P*	HR	95% CI	*P*	HR	95% CI	*P*
Ln OPG	1.87	1.34–2.61	<0.001	1.57	1.08–2.28	0.017	1.16	0.77–1.73	0.481
Age,per 1-year increase	1.04	1.03–1.06	<0.001	1.05	1.02–1.07	<0.001	1.03	1.01–1.05	0.003
BMI, per 1-unit increase	0.93	0.90–0.97	<0.001	0.95	0.92–0.98	0.004	0.95	0.91–0.98	0.003
Creatinine clearance, mL/min	0.98	0.98–0.99	<0.001						
Systolic BP, mmHg	0.99	0.98–1.00	0.022	0.99	0.98–1.00	0.002	0.99	0.98–1.00	0.002
NYHA IV vs II/III	1.87	1.29–2.71	0.001						
History of HF	2.03	1.39–2.95	<0.001						
Atrial fibrillation	1.72	1.18–2.51	0.005						
History of COPD	2.17	1.46–3.20	<0.001	2.00	1.33–3.00	<0.001	2.02	1.33–3.08	<0.001
Ln NT-proBNP	1.39	1.24–1.56	<0.001				1.18	1.02–1.37	0.023
Ln hs-cTnT	1.65	1.41–1.94	<0.001				1.36	1.09–1.68	0.005
Ln CRP	1.10	0.99–1.22	0.08				0.99	0.88–1.12	0.923

OPG osteoprotegerin; BMI body mass index; BP blood pressure; COPD chronic obstructive pulmonary disease; CRP c-reactive protein; hs-cTnT high sensitive cardiac troponin T; NT-proBNP N-terminal pro b-type natriuretic peptide; Prefix Ln for variables transformed by natural logarithm.

^a^Table shows only clinical variables significantly associated with mortality in univariable analysis and biomarkers.

^b^The multivariable analysis include clinical variables associated with all-cause mortality in univariable analysis, and that was kept in the model after reduction with backward selection and p<0.05 as stopping criterion. OPG was not included in the selection process.

^c^Multivariable analysis including the variables in ^b^ and biomarkers.

OPG correlated relatively strongly with age in this cohort (ρ = 0.47; p < 0.001), and the association between OPG and mortality was attenuated after adjusting for age in a multivariable model. However, in the clinical multivariable model, (i.e. without other cardiac biomarkers) the association between OPG and mortality was significant. To assess a potential impact of age on the prognostic value of OPG, we dichotomized age at the median (72.5y) and found that OPG levels were associated with mortality in the lower age group (HR: 3.22 (1.66–6.24); p<0.001) but not in the higher age group (HR: 1.10 (0.74–1.65); p = 0.63). However, no significant interaction in the original multivariable model was observed.

In the subgroup of patients with acute heart failure (n = 139), OPG levels were significantly associated with mortality in crude analyses (HR: 1.70 (1.09–2.66); p = 0.02) (Fig 3B). In contrast, in patients with acute dyspnoea of other causes, OPG levels were not associated with mortality (HR: 1.48 (0.82–2.62); p = 0.19). In 84 of these patients the index diagnosis of the hospital stay was acute exacerbation of COPD and in this subgroup of patients OPG was not associated with mortality (HR: 1.47 (0.68–3.16); p = 0.33) (Fig 3C).

## Discussion

The most important findings of this study of patients admitted to hospital for acute dyspnoea are that (1) OPG levels are significantly higher in patients with acute HF than in patients with acute dyspnoea of other causes, (2) OPG levels are associated with the diagnosis of acute heart failure independently of age, gender, indices of renal function and comorbidities associated with increased OPG levels, but not after established cardiac biomarkers are added to the model, and (3) OPG levels are associated with all cause mortality in patients with acute dyspnoea independently of clinical variables but not after including NT-proBNP and hs-cTnT in the multivariable model. Notably, the association between OPG and mortality was evident in the subgroup of patients with acute HF, but not in those with dyspnoea caused by non-cardiac causes. Taken together, these findings suggest that OPG is not an unspecific marker of inflammation and disease in patients with acute dyspnoea, but associated with cardiac pathophysiology.

### Is OPG a clinically useful biomarker in acute heart failure?

To our knowledge this is the first study to address the diagnostic and prognostic properties of OPG in an unselected population of patients admitted to hospital with acute dyspnoea. In this moderately large study, OPG levels were associated with mortality in the entire acute dyspnoea cohort, but this association was not independent of NT-proBNP or hs-cTnT. Recently Aramburu-Bodas et al. found OPG levels in 177 patients with acute heart failure and preserved EF (EF ≥ 45%), to be independently associated with 1-year mortality from all causes, even after adjusting for NT-proBNP [14]. However, in that study OPG levels were not independently associated with the composite endpoint all-cause mortality and readmission for HF within 1 year. Friões et al. evaluated OPG measured at discharge in 338 patients admitted for acute HF, and found that OPG was an independent predictor of the composite end point all cause mortality and readmission to hospital within 6 months [15]. They did not evaluate all-cause mortality separately. The results of those two studies are not directly comparable to this study, since we evaluated OPG in a cohort including both patients with and without acute HF, and half of the acute HF patient in this study had LVEF<40%. Moreover we did not evaluate the prognostic value of measuring OPG at discharge. In contrast to this study, Aramburu-Bodas et al. excluded patients with a possible increase in acute phase reactants (e.g. infection, active inflammatory disease) and patient using steroids or other hormones and thereby removing factors that may influence OPG levels. There are many potential stimuli that could impact on circulating OPG levels in the setting of acute dyspnoea. For instance, the time point of OPG measurement may be of importance for the prognostic evaluation, although we did not find that OPG levels changed during hospital stay in the 45 acute HF patients were such measurements were available. Larger studies are needed to explain the apparent discrepancies in results of these three studies.

In the current study a main goal was to evaluate the usefulness of measuring OPG in an unselected acute dyspnoea population. In order to evaluate the clinical usefulness of a biomarker, it needs to be tested in a close to real life scenario. We did not find that OPG added diagnostic and prognostic information beyond that already obtained from the established cardiac biomarkers hs-cTnT and NT-proBNP. Although Aramburu-Bodas et al. found a statistically independent association between OPG and mortality in a Cox proportional hazards model [14], reclassification analysis did not show improvement when OPG was added to the model in that study, which supports our results of a more limited clinical utility for OPG in patients with acute dyspnoea. Hence, we believe our results are in concordance with previous results by other groups and that the discrepancy regarding the results of the Cox models may relate to the more narrow selection of participants for the Aramburu-Bodas study.

### Is OPG a cardiac biomarker?

Although our findings do not support OPG as a useful tool for decision-making in the emergency department, our findings suggest that OPG is associated with the pathophysiology of heart failure. OPG was higher in patients with acute HF and was only related to mortality in the patients with acute HF as the index diagnosis. In addition, OPG correlated relatively strongly with NT-proBNP and hs-cTnT. In contrast, in patients with a non-cardiac cause of dyspnoea, OPG was not associated with mortality. Moreover in the subgroup of patients with acute exacerbation of COPD, no association between OPG and mortality was observed. This suggests that systemic OPG is not a biomarker of increased generalised inflammation and is not a useful tool for risk prediction in patients with acute exacerbation of COPD, although the OPG/RANKL/RANK system might be regulated in COPD [16].

The balance between circulating levels of RANKL and OPG have been linked to the development of osteoporosis in COPD [17]. We found that a history of COPD was independently associated with lower OPG levels in this cohort. This finding is in accordance with a previous study by Eagan et al. where OPG levels were lower in 408 patients with stable COPD than in 231 controls after adjusting for confounding factors [18]. OPG levels were higher in sputum of patients with COPD [16], and recombinant OPG stimulated MMP-9 activity in sputum macrophages in vitro, linking OPG to local inflammation and tissue destruction in the lungs. In that study however, OPG sputum levels were not correlated with circulating systemic levels of OPG [16]. Whether lower systemic levels of OPG in patients with COPD is due to treatment with corticosteroids, other specific treatments of COPD or other factors related or unrelated to COPD is unclear. However in the study by Eagan et al. OPG levels were higher in COPD patients with comorbid heart disease, including both heart failure and coronary heart disease [18], linking circulating systemic OPG levels to heart disease in patients with COPD. Moreover, in patients with other pulmonary diseases including obstructive sleep apnoea (OSA) OPG levels were higher in patients with than without cardiovascular disease [19].

In early studies, the OPG/RANKL/RANK system was linked to vascular calcification [20] and the presence and extent of coronary artery disease in patients with stable chest pain [4]. In the current study of patients with acute HF, OPG levels did not differ between patients with and without a history of CAD, suggesting that circulating OPG levels in patients with acute HF are influenced by factors other than the extent of CAD. Invasive coronary angiography was not routinely performed in these patients, so we cannot rule out that some patients have been misclassified concerning the presence of CAD. However, the present observations are in accordance with our finding in 1,229 patients with chronic HF participating in the GISSI-HF study [7], where HF of ischemic origin was not independently associated with OPG levels. It is also in accordance with an early experimental study in rats with post infarction heart failure, where OPG gene expression was upregulated in rats with heart failure, both in the ischemic and non-ischemic part of the myocardium [6].

In accordance with many previous studies [8,21,22], OPG levels were strongly correlated with ageing in this cohort. In a sub-group analysis we found the association between OPG and prognosis to be significant only in younger patients. Given that OPG levels increase strongly with age, this observation is compatible with the theory that circulating OPG levels are linked to heart failure, but that in older individuals other sources may contribute more to circulating OPG and obscure an association between OPG levels, myocardial function and adverse prognosis.

### Strengths and limitations

This study has both strengths and limitations that merit attention. Strengths include the unselected patient group and that the diagnosis of heart failure was adjudicated by an endpoint committee. Limitations include lack of echocardiography in some patients and that cardiovascular mortality was not evaluated. Finally, the ACE2 study is a moderately sized, single centre study and the results must be validated in other cohorts.

## Conclusion

We have shown that OPG is elevated in patients with acute dyspnoea due to heart failure compared to dyspnoea of other causes, but OPG failed to add independent prognostic or diagnostic information to established cardiac biomarkers. Accordingly, despite a statistical association with outcome OPG does not seem to represent a clinically useful tool for the ED physician working with acute HF patients. Still, our findings support investigations to further elucidate a possible pathophysiological involvement of the OPG/RANKL/RANK system in heart failure development.

## Abbreviations

OPG: osteoprotegerin
MMP: matrix metalloproteinase
ACE 2 Study: Akershus Cardiac Examination 2 Study
NT-proBNP: N-terminal pro-B-type natriuretic peptide
HF: heart failure
Q: quartile
AUC: area under the curve
CI: confidence interval
ED: Emergency Department
AMI: acute myocardial infarction
CAD: coronary artery disease
BMI: body mass index
LVEF: left ventricular ejection fraction
COPD: chronic obstructive pulmonary disease
NYHA: New York Heart Association
ROC: receiver-operating characteristics

## References

[1] Maisel AS, Daniels LB (2012) Breathing not properly 10 years later: what we have learned and what we still need to learn. J Am Coll Cardiol 60: 277–282. 10.1016/j.jacc.2012.03.057 22813603

[2] Weintraub NL, Collins SP, Pang PS, Levy PD, Anderson AS, Arslanian-Engoren C, et al (2010) Acute heart failure syndromes: emergency department presentation, treatment, and disposition: current approaches and future aims: a scientific statement from the American Heart Association. Circulation 122: 1975–1996. 10.1161/CIR.0b013e3181f9a223 20937981

[3] Braunwald E (2013) Heart Failure. JACC: Heart Failure 1: 1–20. 10.1016/j.jchf.2012.10.002 24621794

[4] Jono S, Ikari Y, Shioi A, Mori K, Miki T, Hara K, et al (2002) Serum osteoprotegerin levels are associated with the presence and severity of coronary artery disease. Circulation 106: 1192–1194. 12208791 10.1161/01.cir.0000031524.49139.29

[5] Ueland T, Jemtland R, Godang K, Kjekshus J, Hognestad A, Omland T, et al (2004) Prognostic value of osteoprotegerin in heart failure after acute myocardial infarction. J Am Coll Cardiol 44: 1970–1976. 15542278 10.1016/j.jacc.2004.06.076

[6] Ueland T, Yndestad A, Oie E, Florholmen G, Halvorsen B, Froland SS, et al (2005) Dysregulated osteoprotegerin/RANK ligand/RANK axis in clinical and experimental heart failure. Circulation 111: 2461–2468. 15883214 10.1161/01.CIR.0000165119.62099.14

[7] Roysland R, Masson S, Omland T, Milani V, Bjerre M, Flyvbjerg A, et al (2010) Prognostic value of osteoprotegerin in chronic heart failure: The GISSI-HF trial. Am Heart J 160: 286–293. 10.1016/j.ahj.2010.05.015 20691834

[8] Ueland T, Dahl CP, Kjekshus J, Hulthe J, Bohm M, Mach F, et al (2011) Osteoprotegerin predicts progression of chronic heart failure: results from CORONA. Circ Heart Fail 4: 145–152. 10.1161/CIRCHEARTFAILURE.110.957332 21216833

[9] Lippi G, Cervellin G (2014) Risk assessment of post-infarction heart failure. Systematic review on the role of emerging biomarkers. Crit Rev Clin Lab Sci 51: 13–29. 10.3109/10408363.2013.863267 24410541

[10] Rosjo H, Dahl MB, Jorgensen M, Roysland R, Brynildsen J, Cataliotti A, et al (2015) Influence of Glycosylation on Diagnostic and Prognostic Accuracy of N-Terminal Pro-B-type Natriuretic Peptide in Acute Dyspnea: Data from the Akershus Cardiac Examination 2 Study. Clin Chem. 10.1373/clinchem.2015.239673 26056354

[11] McMurray JJ, Adamopoulos S, Anker SD, Auricchio A, Bohm M, Dickstein K, et al (2012) ESC Guidelines for the diagnosis and treatment of acute and chronic heart failure 2012: The Task Force for the Diagnosis and Treatment of Acute and Chronic Heart Failure 2012 of the European Society of Cardiology. Developed in collaboration with the Heart Failure Association (HFA) of the ESC. Eur Heart J 33: 1787–1847. 10.1093/eurheartj/ehs104 22611136

[12] Global Initiative for Chronic Obstructive Lung Disease. Global Strategy for the Diagnosis, Management and Prevention of COPD, Revised 2011.

[13] Cockcroft DW, Gault MH (1976) Prediction of creatinine clearance from serum creatinine. Nephron 16: 31–41. 1244564 10.1159/000180580

[14] Aramburu-Bodas O, Garcia-Casado B, Salamanca-Bautista P, Guisado-Espartero ME, Arias-Jimenez JL, Barco-Sanchez A, et al (2015) Relationship between osteoprotegerin and mortality in decompensated heart failure with preserved ejection fraction. J Cardiovasc Med (Hagerstown) 16: 438–443. 25469731 10.2459/JCM.0000000000000229

[15] Frioes F, Laszczynska O, Almeida PB, Silva N, Guimaraes JT, Omland T, et al (2015) Prognostic Value of Osteoprotegerin in Acute Heart Failure. Can J Cardiol. 10.1016/j.cjca.2015.04.003 26143141

[16] To M, Ito K, Ausin PM, Kharitonov SA, Barnes PJ (2011) Osteoprotegerin in sputum is a potential biomarker in COPD. Chest 140: 76–83. 10.1378/chest.10-1608 21127170

[17] Sarkar M, Bhardwaj R, Madabhavi I, Khatana J (2015) Osteoporosis in chronic obstructive pulmonary disease. Clin Med Insights Circ Respir Pulm Med 9: 5–21. 10.4137/CCRPM.S22803 25788838 PMC4358421

[18] Eagan TM, Ueland T, Wagner PD, Hardie JA, Mollnes TE, Damas JK, et al (2010) Systemic inflammatory markers in COPD: results from the Bergen COPD Cohort Study. Eur Respir J 35: 540–548. 10.1183/09031936.00088209 19643942

[19] Kosacka M, Piesiak P, Porebska I, Jankowska R (2015) Correlations between osteoprotegerin serum levels and body composition parameters in patients with sleep apnea syndrome and the possible influence on cardiovascular risk. Rev Port Pneumol (2006) 21: 239–244. 25926255 10.1016/j.rppnen.2015.01.002

[20] Bucay N, Sarosi I, Dunstan CR, Morony S, Tarpley J, Capparelli C, et al (1998) osteoprotegerin-deficient mice develop early onset osteoporosis and arterial calcification. Genes Dev 12: 1260–1268. 9573043 10.1101/gad.12.9.1260 PMC316769

[21] Yano K, Tsuda E, Washida N, Kobayashi F, Goto M, Harada A, et al (1999) Immunological characterization of circulating osteoprotegerin/osteoclastogenesis inhibitory factor: increased serum concentrations in postmenopausal women with osteoporosis. J Bone Miner Res 14: 518–527. 10234572 10.1359/jbmr.1999.14.4.518

[22] Abedin M, Omland T, Ueland T, Khera A, Aukrust P, Murphy SA, et al (2007) Relation of osteoprotegerin to coronary calcium and aortic plaque (from the Dallas Heart Study). Am J Cardiol 99: 513–518. 17293196 10.1016/j.amjcard.2006.08.064

